# Complexation of DNA with Thermoresponsive Charged Microgels: Role of Swelling State and Electrostatics

**DOI:** 10.3390/gels8030184

**Published:** 2022-03-17

**Authors:** Julia Maldonado-Valderrama, Yan Yang, Maykel Jiménez-Guerra, Teresa del Castillo-Santaella, José Ramos, Alberto Martín-Molina

**Affiliations:** 1 Departamento de Física Aplicada, Universidad de Granada, Campus de Fuentenueva sn, 18071 Granada, Granada, Spain; julia@ugr.es (J.M.-V.); yan137@outlook.com (Y.Y.); maykeljg14@gmail.com (M.J.-G.); tdelcastillo@ugr.es (T.d.C.-S.); 2 Excellence Research Unit “Modelling Nature” (MNat), Universidad de Granada, 18071 Granada, Granada, Spain; 3 IQLIT Emulsiones Poliméricas S.L.U., Autovía Tarragona-Salou Km 3,8., 43110 La Canonja, Tarragona, Spain; j.ramos@iqoxe.com; 4 Instituto Carlos I de Física Teórica y Computacional, Universdad de Granada, 18071 Granada, Granada, Spain

**Keywords:** DNA, microgel, monolayer, hydrodynamic diameter, electrophoretic mobility, compression isotherms, surface pressure, surface tension

## Abstract

Micro- and nanogels are being increasingly used to encapsulate bioactive compounds. Their soft structure allows large loading capacity while their stimuli responsiveness makes them extremely versatile. In this work, the complexation of DNA with thermoresponsive microgels is presented. To this end, PEGylated charged microgels based on poly-N-isopropylacrylamide have been synthesized, allowing one to explore the electrostatics of the complexation. Cationic microgels complexate spontaneously by electrostatic attraction to oppositely charged DNA as demonstrated by electrophoretic mobility of the complexes. Then, Langmuir monolayers reveal an increased interaction of DNA with swollen microgels (20 °C). Anionic microgels require the presence of multivalent cations (Ca^2+^) to promote the complexation, overcoming the electrostatic repulsion with negatively charged DNA. Then again, Langmuir monolayers evidence their complexation at the surface. However, the presence of Ca^2+^ seems to induce profound changes in the interaction and surface conformation of anionic microgels. These alterations are further explored by measuring adsorbed films with the pendant drop technique. Conformational changes induced by Ca^2+^ on the structure of the microgel can ultimately affect the complexation with DNA and should be considered in the design. The combination of microstructural and surface properties for microgels offers a new perspective into complexation of DNA with soft particles with biomedical applications.

## 1. Introduction

Stimuli-responsive microgels are attracting increasing interest in the field of nanomedicine due to the recent development of viruses and artificial platelets [1,2,3]. Microgels are micrometer-sized cross-linked polymeric networks containing a large amount of water inside. Microgels provide a similar microenvironment to that of biological tissues, thus improving their biocompatibility. At present, microgels are perfect candidates for drug encapsulation and release, mainly due to: (i) their higher colloidal stability, compared to micelles and vesicles, which is achieved through the action of crosslinker in their structure; (ii) the stimuli responsiveness induced by different conditions such as pH, temperature, light, and the presence of enzymes or proteins, which can cause significant changes in the characteristics of microgels; and (iii) the control of particle size and charge that can be exerted through the choice of the type and amount of crosslinker and initiators.

One of the most investigated stimulus-response strategies used in oncology is that based on thermally induced drug release [4]. The temperature response is governed by an abrupt change in the properties of the vehicle in such a way that it induces drug release in response to local temperature variation. Ideally, temperature-sensitive carriers retain cargo at physiological temperature (~37 °C), but rapidly release the drug into the locally heated tumor (~40–42 °C). The challenge in designing temperature-sensitive materials is the use of particles that are biocompatible and show sufficient sensitivity in responding to such slight changes close to the physiological temperature. In this regard, liposomes are the most used bioparticles as carriers of gene material [5,6,7]. However, they require a relatively low transfection rate to avoid problems arising from the cytotoxicity associated with these systems. For this reason, nano- and microgels are currently an effective alternative to liposomes, largely due to their great versatility [8,9,10,11,12,13,14,15,16]. In addition, DNA-microgel conjugates can also be used for the development of DNA-based bioassays and biosensors [17,18], as well as for immobilizing oligonucleotides and plasmid DNA in cationic nanogels to protect them from enzymatic degradation [13,15,19,20,21,22,23,24].

In general, the colloidal stability of nano- and microgels must be carefully considered together with their ability to release the encapsulated cargo to achieve successful transport and delivery [13,25]. Moreover, an improved understanding of the physicochemical properties of the gene complexes contributes considerably to the increase in their rates of transfection. In particular, temperature plays a crucial role, as already mentioned above. Accordingly, thermoresponsive microgels based on poly-N-isopropylacrylamide (PNIPAM) appear as good candidates in gene therapy, considering that they present a volume phase transition temperature (VPTT) around the physiological temperature. Namely, they transit from a swollen state (at temperatures below the VPTT) to a shrunken or collapsed state (at temperatures above the VPTT), caused by hydrophobic interactions [26]. Indeed, other characteristics of the microgels and gene material that will influence the different gene transfer strategies are their charge, size, and shape. The soft nature of microgels allows them to transit through pores that are more than 10 times smaller than their size upon application of a force of the order magnitude of the renal filtration pressure [27]. Furthermore, their small and tunable sizes may increase the blood circulation time after administration, and also their large surface area offers a suitable space for functionalization and bioconjugation [9]. Indeed, Wang and Wu showed that transport of gene material across the blood-brain barrier is enhanced thanks to the flexible design of nano- and microgels that allows them to load either complex genes or siRNA for the treatment of neurological diseases [15,28].

In general, polymeric nano- and microgels must contain site-specific cationic entities to spontaneously attract negatively charged gene molecules. Moreover, they can be further conjugated by specific targeting to improve the transfection of gene material into cells and promote their stability under physiological conditions [9,22]. However, anionic nano- and microgels could also be used as gene vectors if multivalent cations are used as mediators between likely charged systems. This strategy has already been employed for the case of anionic lipid-DNA complexes [5,29,30,31]. In this sense, Gelissen et al. have studied a model system of polyampholyte PNIPAM-based microgels with a cationic core and an anionic shell, and they have demonstrated that the presence of a shell still enables efficient uptake and release of polyelectrolytes into the cationic core [32]. Therefore, electrostatics plays an important role in the DNA-microgel self-assembly, which is not always easy to explain.

Apart from the physicochemical properties of the gene complexes, the transfection efficiency of gene complexes depends on their optimal formulation, which in turn is a function of the interaction between the carrier and the gene material. Under this scenario, Langmuir monolayers are a versatile and well-established methodology to elucidate the structural and mechanical properties of biomolecules interacting with different systems [33,34]. In this sense, microgel particles at interfaces are currently being intensively studied for their potential use as emulsion stabilizers [35,36,37,38] as well as for cell adhesion and for haptics as tactile feedback devices [39,40,41]. However, as far as we know, there are no studies in which Langmuir monolayers have been used to study the complexation of DNA by microgels at the air-water interface. Therefore, the main goal of this work is to investigate the interaction of DNA with PNIPAM-based microgel monolayers at the air-water interface. In particular, calf thymus DNA as the gene material and PNIPAM-based microgels synthesized in the presence of poly(ethylene glycol)methacrylate (PEG) as a macro-comonomer are investigated. The use of PEG in the synthesis is twofold: on the one hand, it enhances the biodegradability of the PNIPAM-based microgels [42], and on the other hand, the PEG chains strongly increase the colloidal stability of microgels in electrolyte solutions at high temperatures [43]. In addition, cationic and anionic thermoresponsive microgels (termed mGel+ and mGel−, respectively) are used to evaluate the impact of electrostatic interactions at different temperatures, i.e., swelling state. To this end, a Langmuir film balance is used to measure different microgel spread films. Next, Gibbs-adsorbed films are formed at the air-water interface to investigate in more detail the surface conformation of some selected systems. Hence, dynamic adsorption and interfacial dilatational rheology of mGel-adsorbed films at the air-water interface are measured to explore the conformational changes induced by the electrolyte in the surface properties of microgel particles. Findings are also discussed considering the hydrodynamic diameter and electrophoretic mobility of microgels as they complexate with DNA in solution. The complexation takes place spontaneously between oppositely charged mGel+ and DNA and is mediated by electrolytes for similarly charged mGel− and DNA. The results presented herein offer a new perspective on the physiochemistry underlying the complexation of soft particles and DNA which can have applications in the understanding and design of novel gene delivery systems.

## 2. Results and Discussion

As mentioned above, two types of PEGylated microgels based on PNIPAM with opposite net charge have been used to explore the complexation mechanisms and interactions with DNA. Microgels were synthesized by IQLIT and were kindly provided. The recipe used is detailed in Section 4.2 and the composition is displayed in Table 1. The acronyms in the table account for the following compounds: N-Isopropylacrylamide (NIPAM), Methoxypolyethylene glycol 750-methacrylatem 50% in water (MPEG750MA), methacrylic acid (MAA), [2-(Methacryloyloxy)ethyl]trimethylammonium chloride (MATMAC), Ethylene glycol dimethacrylate (EGDMA), Sodium persulfate (NaPS), and 2,2′-Azobis(2-methylpropionamidine)dihydrochloride (V-50).

**Figure 1:**
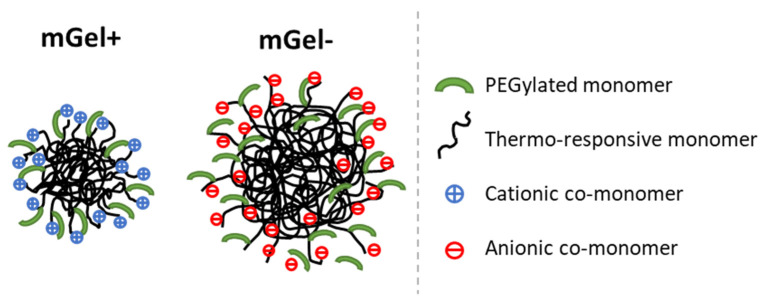
Schematic diagram of the microgel particles synthetized following the procedure detailed in Section 4.2. Details on the components are displayed in Table 1.

Figure 1 shows a schematic illustration of the synthesized microgels, one anionic and one cationic, with PEGylated monomer inserted in the shell. The larger size of anionic microgel is given by the slower rate of radical formation compared with the faster rate in the case of cationic initiator. This means that more particles are formed in the case of cationic system providing smaller particles [44]. In this study, first, the complexation of cationic microgel (mGel+) with oppositely charged DNA is investigated, which is expected to proceed spontaneously by electrostatic attraction. Second, the complexation of anionic microgels (mGel−) with similarly charged DNA, which proceeds mediated by multivalent cations, is presented. In both cases, experiments in bulk and at the air-water interface are discussed in a combined manner to highlight the different interactions. Finally, the effect of multivalent cations on the surface conformation of mGel− is further analyzed with a pendant drop film balance.

### 2.1. DNA-Cationic Microgel Complexes

#### 2.1.1. Bulk Behavior

**Figure 2:**
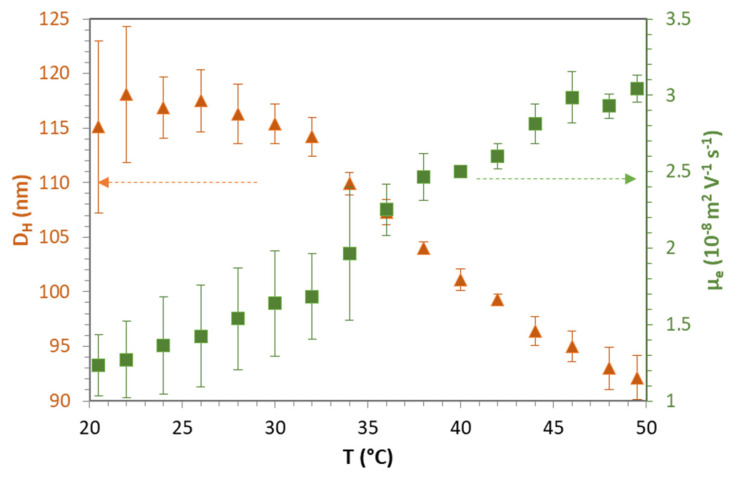
Hydrodynamic diameter (orange triangles) and electrophoretic mobility (green squares) of mGel+ in ultrapure water, as a function of temperature. Values plotted are mean of three independent measurements with standard deviation.

The size and electrokinetic data of mGel+ in pure water as a function of the temperature of the media are shown in Figure 2. In particular, the diameter of the microgel particles (D_h_) and their electrophoretic mobility (µ_e_) were measured for temperatures ranging between 20 °C and 50 °C. The experiments were always performed from low to high temperatures, as PNIPAM generally shows no hysteresis [45]. As can be seen in Figure 2, D_h_ decreases from values around 117 nm for temperatures below 30 °C, to diameters smaller than 100 nm for temperatures above 40 °C. A more precise value for the VPTT can be obtained if we analyze the slope at a point on the curve to give a value of 36 °C. Since the typical VPTT for pure PNIPAM microgels is around 32 °C, it indicates that the PEG does not have much influence on the swelling behavior of the microgels. This feature has recently been reported for PEGylated PNIPAM microgels [43]. The positive sign of µ_e_ is due to the cationic comonomer (MATMAG) and initiator (V-50) used in the synthesis of mGel+ (Table 1). µ_e_ increases with the temperature by almost tripling its value when microgels shift from swollen to shrunken (Figure 2).

A similar trend has been observed for charged thermoresponsive PNIPAM-based microgels [45,46,47]. This increase of µ_e_ with the temperature is usually explained by a reduction of the friction of the particles when they shrink and also in terms of the effective charge of the particles. If the temperature induces a reduction in the particle size, its effective charge increases. In fact, effective charge of microgels is assumed to be proportional to its surface electrostatic potential which, in turn, increases with the temperature [48].

**Figure 3:**
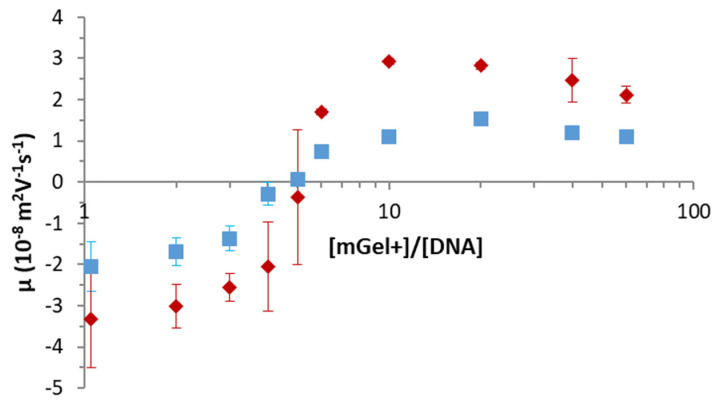
Electrophoretic mobility of DNA/mGel+ complexes at different [DNA]/[mGel+] concentration ratios for swollen (20 °C, blue squares) and shrunken (50 °C, red rhomboids) microgels. The concentration of DNA is fixed at 0.025 g L^−1^ and the concentration of mGel+ varies between 0.025 and 1.5 g L^−1^. Values plotted are the mean of three independent measurements with standard deviation.

Once the particles of mGel+ in pure water are characterized, the electrokinetic behavior of the DNA/mGel+ complexes is evaluated. To this end, the DNA concentration of [DNA] = 0.025 g L^−1^ is fixed whereas the microgel concentration varies between 0.025 and 1.5 g L^−1^ as plotted in Figure 3. The electrophoretic mobility of DNA was measured, obtaining values of −(3.8 ± 2.2) µm cm V^−1^ s^−1^. This value was practically independent of temperature (results not shown). Therefore, it can be assumed that the negative magnitude of μe obtained at very low [mGel+]/[DNA] ratios (Figure 3) is mainly due to free DNA in the sample. The experimental data of µ_e_ as a function of the [DNA]/[mGel+] ratio for both temperatures exhibit sigmoidal curves that can be divided into three regions: (i) a region where the complexes show a net negative µ_e_; (ii) a region where the isoelectric point (value of [DNA]/[mGel+] at which µ_e_ = 0) is reached and a reversal of the sign of µ_e_ occurs; (iii) a region of net positive and almost constant value of µ_e_ that matches that of pure microgels at temperatures 20 °C and 45 °C, respectively (Figure 2). This behavior was previously found and studied in great detail for cationic liposome/DNA complexes (lipoplexes) [49,50,51,52,53]. However, it has not been reported specifically for nano- or microgel/DNA complexes, but only for comparable systems. The closer examples found in the literature are Tamura et al., who reported the reversal of the ζ-potential of complexes formed by PEGylated polyamine nanogels composed of a chemically cross-linked poly[2-(N,N-diethylaminoethyl)methacrylate] (PDEAMA) core surrounded by PEG-tethered chains and siRNA [20]. Likewise, Kleinen and Richtering also provide electrophoresis measurements to investigate the charge inversion of polyelectrolyte-microgel complexes formed by cationic poly(diallyldimethylammonium chloride), PDADMAC, and anionic PNIPAM microgels [54,55]. More recently, Sennato et al. have shown that when PNIPAM microgels are collapsed, they strongly interact with oppositely charged polyelectrolytes and a large charge inversion is observed [56]. In this sense, coarse-grained Monte Carlo simulations have recently been applied to predict the charge inversion previously detected through µ_e_ for nanogel-polyelectrolyte complexes [57]. Therein, it is demonstrated that charge inversion occurs only if the polyelectrolyte charge is large enough [57]. Apart from the reversal observed in the sign of the µ_e_ shown in Figure 3, we can also observe that the isoelectric point of DNA/mGel+ complexes does not depend on the temperature, i.e., the swelling state of the mGel+. Interestingly, the same quantity of DNA is required in both cases to invert the sign of the complexes. Yet, more stable complexes (with larger |µ_e_|) are formed at temperatures above VPTT, in which microgels are shrunken.

#### 2.1.2. Langmuir Spread Films

**Figure 4:**
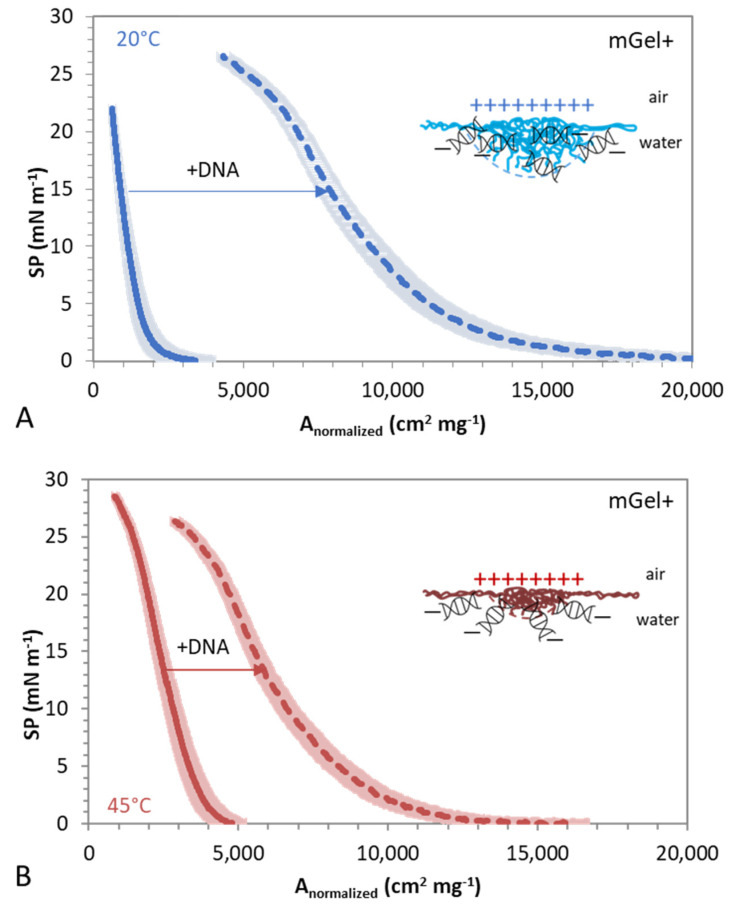
Surface pressure area compression isotherms for mGel+ spread on ultrapure water (solid lines) and on subphase with [DNA] = 0.025 g L^−1^. (**A**) Swollen microgels, 20 °C. (**B**) Shrunken microgels, 45 °C. Values plotted are the mean of three independent measurements with standard deviation in lighter color. Schematic diagram of the surface interaction between DNA/mGel+. DNA illustration is not to scale.

In order to investigate the nature of the interaction of DNA/mGel+, Langmuir films of spread mGel+ particles are formed on pure water subphase and on subphase containing DNA (Figure 4). Aqueous solutions of mGel+ were spread on the surface of an aqueous solution in a Langmuir–Pockels film balance. Herein, the total surface area (A) is reduced by lateral compression of movable barriers while measuring the surface pressure (SP), providing the SP-A isotherms (Figure 3). The compression SP-A isotherms are recorded on pure water subphase and on subphase containing DNA. In both cases, the absence of surface activity of DNA was checked before every experiment, proving SP < 0.2 mN m^−1^ within the whole compression range.

The objective here is to evaluate the influence of the swelling state, or softness, of the microgels on the surface phase behavior upon compression, the interaction regimes, and complexation with DNA to compare and complement the complexation found in bulk (Figure 2). Figure 4A,B show the resulting surface pressure area (SP) isotherms of swollen (25 °C) and shrunken (45 °C) mGel+ in the presence and absence of DNA in the subphase. The surface particles show different interaction regimes as the interparticle distance diminishes by lateral compression of the monolayer. In this way, analysis of SP-A isotherms highlights the different interaction regimes of mGel+ and DNA/mGel+ at the surface layer (and the polymer chains that form them), as the surface particles approach.

On a pure water subphase, the lateral compression of mGel+ produces a uniform increase of the SP which proceeds as the surface area is compressed until collapse. This shape is similar for mGel+ above (Figure 4A) and below (Figure 4B) the VPPT. It is generally accepted that microgel particles spread at the surface, flattening the shells, while the crosslinked core remains in the bulk, adopting the well-known “fried egg structure” [37] as also schematically visualized in the illustration. Microgels undergo different interaction regimes as the interparticle distance decreases upon compression. In a first regime, for large surface areas, microgels behave as a gas of non-interacting particles leading to a negligible SP. Further compression results in a continuous increase of the SP, indicative of the existence of a second regime of interaction, classically denoted as expanded liquid. The SP increases when peripheral shells of flattened microgels come into contact and proceeds with the interpenetration of the shells until collapse, presumably when the size of non-deformed microgels is reached [58]. In summary, the uniform increase of SP obtained below and above the VPPT (Figure 4A,B) is indicative of the existence of a single interaction regime between mGel+ in the swollen and shrunken states corresponding to the interaction of flattened shells until the collapse of the monolayer [59]. The electrostatic repulsion between cores prevents the existence of interaction regimes at closer interparticle distances [46]. The SP-A isotherms obtained for swollen and collapsed mGel+ showed no significant differences, suggesting that in this case, the flattening upon spreading prevails over the temperature-induced swelling state of mGel [60].

Conversely, in the presence of DNA, the obtained SP-A isotherms recorded for shrunken and swollen DNA/mGel+ (Figure 4A,B) show significant differences with respect to those obtained for mGel+. Complexation of DNA/mGel+ has a significant effect on the state of the mGel+ monolayer, which in turn appears to be also clearly influenced by the swelling state of the particles. Interestingly, the shape of the isotherms recorded for DNA/mGel+ below and above the VPTT is similar, as occurred in the absence of DNA. However, the SP-A isotherms of DNA/mGel+ appear to be displaced to higher normalized areas and emerge more expanded than those recorded for mGel+, for both swollen (Figure 4A) and shrunken (Figure 4B) states. These changes suggest a considerable alteration of the state of the spread layer of mGel+, induced by interaction with DNA at the surface. On one hand, the displacement to higher normalized areas indicates that the shells of flattened DNA/mGel+ begin to interact at larger interparticle distances, suggesting an induced larger lateral extension of the mGel+ in the presence of DNA. Similar shifts of the (SP–A) isotherms of cationic lipid monolayer to larger molecular areas, electrostatically induced by DNA, have been reported for salmon sperm DNA interacting with cationic lipid films at the air-water interface [61]. On the other hand, the increased expansion of the SP-A isotherms obtained for DNA/mGel+ suggests an increased deformability of the complex DNA/mGel+. While the mGel+ could be barely compressed, the presence of DNA significantly lengthened the monolayer (Figure 4). The presence of DNA also induced a very slight plateau at high compression ratios, while for single mGel+ the isotherm increases more steeply without forming a plateau. This is attributed to slight desorption of chains at higher compression rates caused again by the increased ability of the DNA/mGel+ complex to deform at the surface. A similar effect was found by decreasing the crosslinking density of microgel particles, which also resulted in increased deformability and extension of spread microgels in SP-A compression isotherms [47]. In the case of interaction with DNA, possibly the DNA strands are able to interpenetrate into the mGel structure which stretches at the surface, hence expanding the monolayer. The long-range electrostatic attraction between DNA and mGel+ is responsible for the diffusion of the DNA molecules towards the interface. However, once the DNA reaches the monolayer, complexation with microgels induces structural changes in the microgel particles that will also depend on the temperature, i.e., on the swelling state. Indeed, the displacement of the monolayer is significantly larger for DNA interacting with swollen mGel+, below the VPTT (Figure 4A) than with shrunken mGel+, above the VPPT (Figure 4B). It can be hypothesized that softer swollen microgels (below VPTT) feature a larger number of dangling chains in their corona, which can locally expose more positive charges. As the fuzzy corona increases the surface area, there could be more anchoring points exposed to the negatively charged DNA, hence improving the interaction. The existence of an additional non-electrostatic interaction based on bio-multiple hydrogen bonding between DNA and PNIPAM is another possibility. In this line, Lee et al. show improved interaction of uncharged PNIPAM and herring sperm DNA at 25 °C at a given ratio [18]. Moreover, the more porous structure of swollen microgel should allow the entanglement of more DNA strands trapped in the mGel+, also accounting for the increased expansion of the monolayer.

### 2.2. DNA-Anionic Microgel Complexes

#### 2.2.1. Bulk Behavior

**Figure 5:**
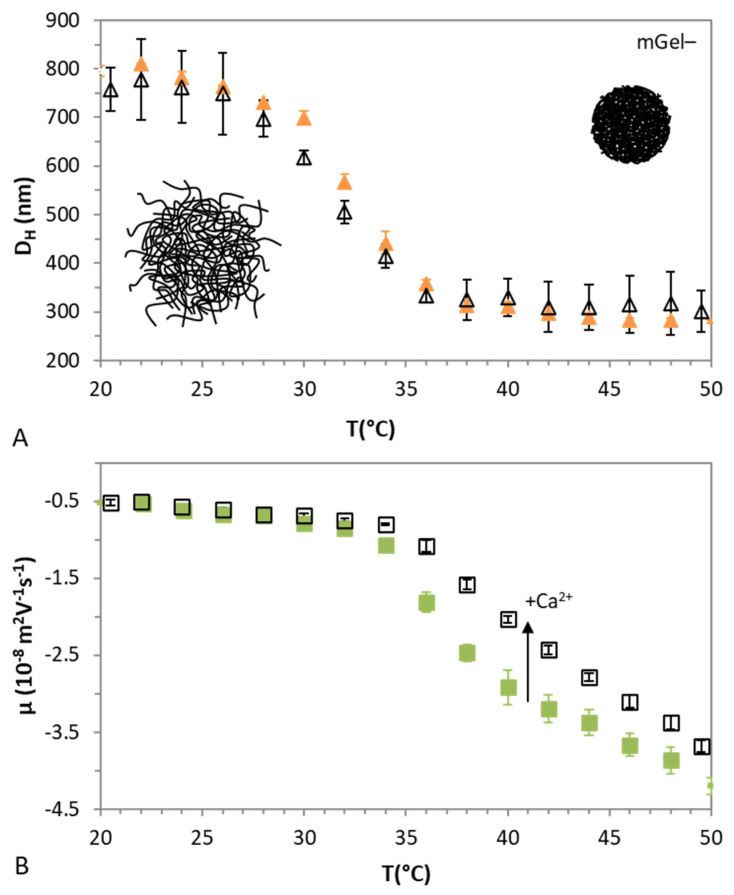
(**A**) Hydrodynamic diameter and (**B**) electrophoretic mobility of mGel− as a function of temperature in water (solid symbols) and in 1 mM Ca(NO_3_)_2_ (open symbols). Values plotted are the mean of three independent measurements with standard deviations.

In general, adsorption of polyelectrolytes onto like-charged surfaces requires the use of oppositely charged entities that act as (electric) bridges between them [62]. For instance, anionic lipid-DNA complexes are usually formed by multivalent cations [5]. In this section, the complexation of DNA with anionic microgels (mGel−) mediated by Ca^2+^ is evaluated. To begin with, the swelling behavior of mGel− in water and in a solution with 1 mM of Ca(NO_3_)_2_ is explored. Figure 5A shows the D_h_ of mGel− as a function of the temperature in both cases. It can be seen that mGel− is bigger than mGel+ as also depicted in Figure 1. Moreover, Figure 5A shows that mGel− exhibits a swelling transition behavior larger than that obtained for the case of mGel+ where the initiator decomposes faster and produces more radicals, forming more initial particles. Diameters around 800 nm and 300 nm are found for low and high temperatures, respectively, whereas their VPTT is close to 33 °C. As can be seen in this figure, the effect of 1 mM of Ca(NO_3_)_2_ on the D_h_ of the microgel is negligible.

Concerning the electrokinetic behavior, Figure 5B shows the µ_e_ of mGel− as a function of the temperature in the presence and absence of electrolyte. Negative mobilities for mGel− are reported because of the anionic comonomer (MAA) and initiator used in the synthesis (NaPS) as shown in Table 1. As expected, the magnitude of µ_e_ (|µ_e_|) increases when microgels shrink. Interestingly, the effect of the cations is now discernible for shrunken mGel− by slightly reducing their effective charge at T > VPTT. From a colloidal point of view, the electrolyte screens the charge of the microgels to some extent. Similar results have been reported for anionic PNIPAM-based microgels as well as anionic PVCL-based microgels in the presence of NaCl, MgCl_2_ and LaCl_3_ [63]. Therein, the authors state that μ_e_ depends on the microgel size, the charge density inside the microgel as well as on the drag coefficient. In addition, these three magnitudes may also depend on the temperature and on the valence of the counter ion. Accordingly, the increase of μ_e_ as the microgel shrinks in the presence of electrolyte as the result of a complex combination of these parameters [63].

At this stage, the complexation of DNA/mGel− cannot be addressed based on electrophoresis measurements as they are both negatively charged. Hence, in order to explore the complexation of DNA with mGel−, it is necessary to move on to the analysis of Langmuir spread films of mGel− and DNA/mGel− as in the previous section. However, several questions arise here: (i) will the DNA affect the surface conformation of mGel− spread films in the absence of Ca^2+^? (ii) Will the presence of Ca^2+^ influence the state and interaction regimes of mGel− spread films? (iii) Will Ca^2+^ be able to attract DNA towards mGel− monolayers? (iv) Will these interactions be affected by the swelling state of mGel−? All these questions will be discussed in the following sections.

#### 2.2.2. Langmuir Spread Films

**Figure 6:**
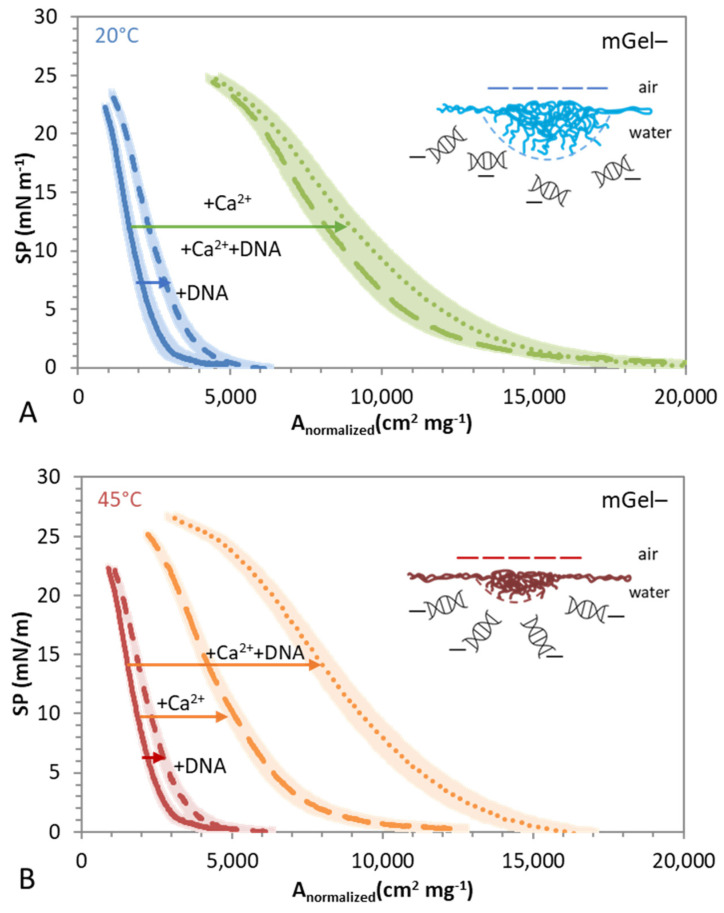
Compression isotherms obtained for swollen mGel− spread on different subphases: water (solid lines), DNA (short dash), Ca(NO_3_)_2_ (long dash), and DNA+Ca(NO_3_)_2_ (dot)_._ [DNA] = 0.025 gL^−1^, [Ca(NO_3_)_2_] = 1 mM. (**A**) Swollen microgels, 20 °C. (**B**) Shrunken microgels, 45 °C. Values plotted are the mean of three independent measurements with standard deviation in lighter color. Schematic diagram of the surface interaction between DNA/mGel− mediated by Ca^2+^. DNA illustration is not to scale.

Following a similar approach to that followed to investigate the interaction of DNA/mGel+ in Figure 4, Langmuir spread films of mGel− are formed now on different subphases, composed of pure water and aqueous solutions containing DNA, Ca(NO_3_)_2_, and DNA+Ca(NO_3_)_2_. In all cases the absence of surface activity of bulk solutions was tested before every experiment by compressing the bare aqueous solution surface and obtaining negligible values of SP. Again, the aim of these experiments was to explore the interactions between Gel– and DNA mediated by the presence of cations (Ca^2+^), with the focus set on the impact of swelling behavior on the possible complexation with DNA. Figure 6A,B show the resulting SP-A compression isotherms obtained for swollen (25 °C) and shrunken (45 °C) mGel− spread on subphases containing pure water, DNA, Ca^2+^, and DNA/Ca^2+^. Again, analysis of the different SP-A isotherms highlights the different interaction regimes of mGel−, DNA/mGel−, DNA/Ca^2+^/mGel+, and Ca^2+^/mGel as the surface area is compressed and the interparticle distance diminishes, hence providing information on the complexation mechanism and the role of Ca^2+^.

On a pure water subphase, it can be inferred again from Figure 6 that mGel− particles spread at the surface and undergo a single transition from non-interacting-particles (negligible SP), at large surface areas, to an interaction of shells. The latter is characterized by a uniform increase of the SP owing to contact and interpenetration of shells of flattened microgels spread as the surface area is compressed. These interaction regimes occur similarly for swollen (Figure 6A) and shrunken (Figure 6B) mGel− as also occurred for mGel+ (Figure 4).

In the presence of DNA, the obtained SP-A isotherms recorded for swollen (Figure 6A) and shrunken (Figure 6B) DNA/mGel− just slightly displace the SP-A isotherm obtained for mGel− (on pure water subphase). This displacement is very small but statistically meaningful and is slightly larger for swollen DNA/mGel+ (Figure 6A). This finding indicates that, contrary to the results obtained for mGel+ in Figure 4, the DNA is not able to produce significant alterations in the state of the monolayer, indicating a minor interaction between DNA and mGel− (Figure 6). This lack of complexation was expected and is most likely due to the electrostatic repulsion between both negatively charged DNA and mGel−. This long-range repulsive interaction prevents the diffusion of DNA towards the mGel− covered surface, hence hindering the complexation, especially for shrunken mGel− (Figure 6B), consistent with the higher electrokinetic mobility in bulk (Figure 5B). Whereas below the VPPT, some DNA strands would be able to interpenetrate into the mGel− structure, stretching to some extent and somewhat expanding the monolayer (Figure 6A), also in agreement with lower electrokinetic mobility measured in bulk for swollen mGel− (Figure 5B). However, the effect is very small as there is not an attractive interaction in any case as occurred for DNA/mGel+. Analogous results were observed for anionic lipid monolayers, in which the SP-A isotherm remained almost invariable under the presence of DNA in the aqueous subphase [29].

Interestingly, in the presence of Ca^2+^ in the subphase, the obtained SP-A isotherms recorded for swollen (Figure 6A) and shrunken (Figure 6B) Ca^2+^/mGel− show very significant differences with respect to those obtained for mGel− and DNA/mGel−. The SP-A isotherms appear to be now displaced to larger normalized areas and are clearly more expanded. Moreover, this displacement is considerably more notable for swollen mGel– (Figure 6A). Indeed, the origins of this displacement are unclear. On one hand, the presence of Ca^2+^ screens the electrostatic repulsion between mGel− particles, hence promoting the spreading and flattening of the shells and also promoting shell interactions and interpenetrations. However, the amount of Ca^2+^ is not enough to fully screen the electrostatic repulsion (Figure 5B). Moreover, the displacement and expansion are significantly higher for swollen mGel− (Figure 6A) in which the electrostatic repulsion was already minimal. A shift of the SP-compression isotherms induced by the addition of salt to the aqueous subphase has recently been reported for the case of anionic monolayers formed by thermoresponsive PNIPAM based on microgels [47]. Therein, the presence of 1 mM of NaNO_3_ also shifted SP-A isotherms towards larger areas for temperatures below and above their VPTT. Nevertheless, the existence of a specific interaction of the mGel− with Ca^2+^ will be explored in more detail below.

A most surprising result arises in the presence of DNA/Ca^2+^ in the subphase, where the obtained SP-A isotherms recorded for DNA/Ca^2+^/mGel− depend primarily on the swelling state of the mGel−. On one hand, below the VPTT (Figure 6A), the SP-A isotherm obtained for swollen DNA/Ca^2+^/mGel− overlaps with that obtained for swollen Ca^2+^/Gel–, showing no significant differences by addition of DNA. On the other hand, above the VPTT (Figure 6B), the SP-A isotherm obtained for shrunken DNA/Ca^2+^/mGel− is substantially expanded and displaced to higher normalized areas. These are unexpected findings that might highlight important differences between the complexation of DNA and mGel−; both might be dependent on the swelling state of the mGel and might be mediated by the presence of Ca^2+^. However, this apparent lack of complexation of DNA/Ca^2+^/mGel− in a swollen state could also be related to limitations of the experimental technique. The amount of Ca^2+^ is not enough to screen the electrostatic repulsion between DNA and shrunken mGel− (Figure 5B). Accordingly, the origin of the larger displacement and expansion of the shrunken Ca^2+^/DNA/mGel possibly arises from interaction with DNA mediated by Ca^2+^, which now allows DNA strands to interpenetrate into the mGel− structure, stretching the surface, hence expanding the monolayer, and improving its deformability. This effect could also be occurring in the swollen DNA but it is hindered by saturation of the interface owing to an already fully expanded conformation of the Ca^2+^/mGel− (Figure 6A and discussion above). At this stage, it was decided to look into the surface conformational changes induced by Ca^2+^ on the mGel− by measuring Gibbs adsorbed layers with pendant drop equipment.

#### 2.2.3. Gibbs Adsorbed Films

**Figure 7:**
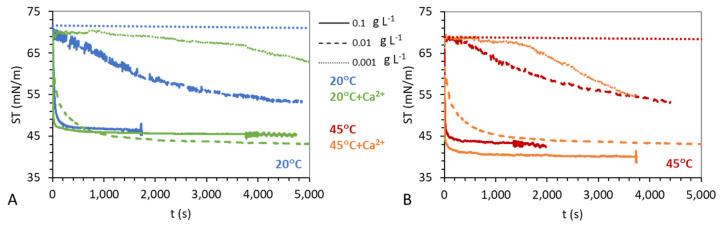
Dynamic surface tension of adsorption of mGel− onto the air-water interface at constant surface area and for different mGel− concentration, temperature and subphase: 0.1 g L^−1^ (solid line), 0.01 g L^−1^ (dashed line), 0.001 g L^−1^ (dotted line). (**A**) Swollen microgels, 20 °C in water (blue), in 1 mM Ca(NO_3_)_2_ (green). (**B**) Shrunken microgels, 45 °C in water (red), in 1 mM Ca(NO_3_)_2_ (orange). Plotted values are mean of at least three experiments, being the standard deviation below ±2 mN/m.

**Figure 8:**
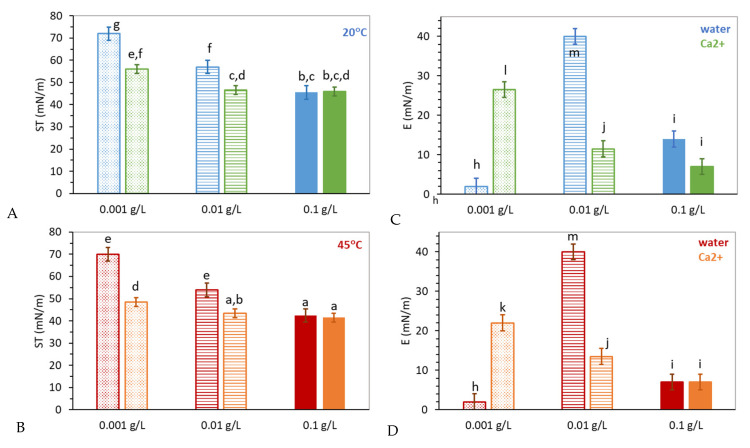
(**A**,**B**) Final surface tension, measured after different time scales from Figure 7, and (**C**,**D**) dilatational elastic modulus (0.1 Hz) reached after adsorption at constant surface area for different mGel− concentration, temperature, and subphase: 0.1 g L^−1^ (solid bar), 0.01 g L^−1^ (striped bar), 0.001 g L^−1^ (dotted bar). (**A,C**) Swollen microgels, 20 °C in water (blue), in 1 mM Ca(NO_3_)_2_ (green). (**B**,**C**) Shrunken microgels, 45 °C in water (red), in 1 mM Ca(NO_3_)_2_ (orange). Plotted values are the mean of at least three experiments and the error bars stand for standard deviation. The statistics study was conducted considering the mean value, deviations, and number of repetitions of each experiment and comparing the final surface tension (a–g) and the dilatational elastic modulus (h–m), independently. Different letters (a–g) and (h–m) indicate significant differences between samples (*p* < 0.05).

The pendant drop surface film balance allows studying the deformation and packing of spontaneously adsorbed microgels. Figure 7 shows the surface tension (ST) of aqueous solutions of mGel− as a function of time for three concentrations (0.1, 0.01, 0.001 g L^−1^). These are measured in water subphase and in subphase containing Ca^2+^, and experiments are also performed below (Figure 7A) and above (Figure 7B) the VPTT to account for the swelling state and under similar conditions to those used in Langmuir films. The ST was measured during different time scales depending on the concentration of the sample since the less concentrated solutions did not always attain a steady state value. Hence, the final values, registered in Figure 8, are considered non-equilibrium states useful for the purpose of assessing the effect of Ca^2+^ on the surface activity of mGel− but do not account for an equilibrium surface film.

The adsorption behavior of microgel particles is peculiar and slightly different to that of linear surfactants or proteins as recognized in the literature [58,64]. According to Tatry et al., different concentrations of mGel converge towards the same ST with faster kinetics as the concentration increases in bulk. In fact, the concentration range in which the system changes from nonadsorbing to adsorbing is very narrow. Figure 7 shows the limiting concentrations found for mGel in pure water and how these are affected by the presence of Ca^2+^.

In pure water subphase, the lowest concentration of mGel− (0.001 g L^−1^) provides a negligible change of SP, for swollen (Figure 7A) and shrunken (Figure 7B) mGel−. This is due to the low amount of particles, high hydrophilicity, and solubility of mGel−, resulting in a low tendency of the particles to migrate to the surface. The intermediate concentration (0.01 g L^−1^) provides a slow decrease of ST which reaches a pseudo plateau offering a non-equilibrium state before saturation of the interface. The highest concentration (0.1 g L^−1^) provides a rapid decrease of the ST until a final value corresponding to a saturated surface. The kinetic evolution of the ST demonstrates the spontaneous adsorption of mGel−, which is faster as the bulk concentration increases and proceeds quite similarly for swollen (Figure 7A) and shrunken microgels (Figure 7B).

In the presence of Ca^2+^ the kinetic evolution of ST is significantly faster for all the concentrations evaluated. All three concentrations provide a significant reduction of the ST and the intermediate concentration (0.01 g L^−1^) provides a saturated surface in the presence of Ca^2+^ while it is now the lowest concentration (0.001 g L^−1^), which provides a non-equilibrium value after 5000 s. In fact, Figure 7 illustrates how the final value of ST reached for mGel− in the presence of Ca^2+^ appears independent of the bulk concentration, while the kinetics depends on bulk concentration. As already commented, this behavior has already been reported for microgel particles and contrasts to that of conventional surfactants where the steady-state value of ST decreases with bulk concentration until the critical micelle concentration [58,64]. This peculiar adsorption kinetics of microgels reveals cooperative behavior and the existence of strong attractive interactions at the interface that promote flattening and expansion of shells upon adsorption. Actually, in the absence of Ca^2+^, the final SP reached is possibly also independent of concentration, but for longer adsorption times (results not shown). Besides, the faster kinetics obtained for mGel− adsorption in the presence of Ca^2+^ agrees with results obtained by Tatry et al., who show that the addition of salt (NaCl) accelerates the kinetics for both charged and uncharged microgels [64]. Given that the amount of mGel is the same, the reason for this faster kinetics should be related to a conformational change induced by Ca^2+^ which may reduce the hydrophilicity of the mGel−.

Figure 8 seeks to compare the properties of a final adsorbed layer obtained in water and in the presence of Ca^2+^ in the subphase, for swollen and shrunken mGel−. This is accomplished by evaluating the final ST attained upon adsorption (Figure 8A,C) and the dilatational elasticity of adsorbed films E (Figure 8B,D). Although for the critical concentrations, the final ST value corresponds to a non-equilibrium plateau, it provides qualitative information. As already mentioned, the final ST obtained for the same concentration of mGel in the presence of Ca^2+^ depends fundamentally on the bulk concentration, i.e., surface coverage. At the lowest concentrations, the final ST attained by mGel− is lower in the presence of Ca^2+^ (Figure 8). By contrast, at higher bulk null concentrations, the surface saturates by flattening of shells and interaction. Hence, the increased adsorption measured at the lowest concentrations could be originated either by more particles reaching the surface or by promoted flattening of the adsorbed mGel−. This would be consistent with an increased local hydrophobicity induced by Ca^2+^. In fact, a large capability of Ca^2+^ to dehydrate anionic lipid membranes has been reported to explain changes in the molecular structure of anionic lipid monolayers [65,66]. In this regard, more recent works demonstrate that Ca^2+^ can also induce structural changes in anionic polyelectrolytes at the air–water interface [67]. In particular, the hydrophobicity of poly(sodium 4-styrenesulfonate) (NaPSS) polyelectrolytes at the air-water interface increases in the presence of Ca^2+^ in the aqueous subphase. This effect of Ca^2+^ on the hydrophobicity of anionic lipids and anionic polyelectrolytes at the surface leads us to hypothesize that a similar effect occurs in monolayers formed by anionic polymeric microgels.

At this stage, the surface dilatational rheology of the surface layer may provide further information of the conformational state of adsorbed mGel− in the presence and absence of Ca^2+^. The surface dilatational elasticity measured in all cases indicated a predominantly elastic behavior, as the elastic modulus was around ten times larger than the loss modulus (results not shown). Accordingly, only the dilatational elasticity (E) is plotted and discussed. In general, it is known than the E of adsorbed films presents a maximum value for mGel− adsorbed layers in water corresponding to the flattened conformation of microgels in which the amount of segments are anchored at the surface and hence where the surface coverage is maximized [58]. In this work, three concentrations have been chosen, accounting for non-adsorbing (0.001 g L^−1^), intermediate/critical (0.01 g L^−1^), and saturated layer (0.1 g L^−1^), for mGel- in water, as explained above. Then again, the effect of Ca^2+^ on the E of the adsorbed layer is analyzed for these three concentrations in Figure 8C,D.

In pure water subphase, the lowest concentration of mGel− (0.001 g L^−1^) shows a null E for swollen (Figure 8C) and shrunken (Figure 8D) mGel− adsorbed layers, corroborating the negligible adsorption (ST) recorded in Figure 7. However, it has already been discussed that the presence of Ca^2+^ promotes the adsorption of mGel− at this lowest concentration. Figure 8 shows that adsorbed mGel− also develops an elastic network in the presence of Ca^2+^, which is more elastic for swollen mGel−, similar to the higher elasticity of swollen microgels in pure water subphase [46].

As the concentration of mGel− increases (0.01 g L^−1^), the increased adsorption is accompanied by a reversed trend on the E which now decreases in the presence of Ca^2+^ and proceeds similarly for swollen (Figure 8C) and shrunken (Figure 8D) mGel−. This indicates that the maximum flattening of mGel− is displaced to lower bulk concentrations in the presence of Ca^2+^. Accordingly, the flattened shells should be more expanded, and interpenetration of shells should also be promoted in the presence of Ca^2+^. The screening of electrostatic repulsion and a specific interaction between Ca^2+^ and mGel could be considered responsible for this effect.

Finally, at the highest concentration of mGel− (0.1 g L^−1^) the lower E suggests the increased interpenetration of shells with respect to less concentrated samples in which the mGel− shells are more expanded. The interpenetration and surface coverage appear similar for shrunken mGel– and slightly different for swollen mGel− in the presence of Ca^2+^. The lower E obtained for swollen mGel− in the presence of Ca^2+^ suggests that interpenetration of flattened shells proceeds at lower bulk concentrations, in turn accounting for a larger extension of mGel− induced by Ca^2+^.

## 3. Conclusions

Electrophoresis experiments confirm the spontaneous complexation of mGel+ by electrostatic attraction with oppositely charged DNA. Then, Langmuir monolayers reveal an increased interaction of DNA with swollen mGel+ (above the VPPT) at the surface, which is measurable by the expansion of SP-A isotherms of DNA/mGel−. Conversely, electrophoresis is no longer practical to address the complexation of DNA and mGel−, which requires the use of multivalent cations (Ca^2+^) to promote complexation overcoming electrostatic repulsion. Then again, Langmuir monolayers evidence that the presence of Ca^2+^ promotes the complexation with DNA, which otherwise barely alters the state of the monolayer as the SP-A isotherms of DNA/mGel− overlap with those of mGel−. However, the complexation of DNA/Ca2^2+^/mGel− is only measurable for shrunken mGel− (above the VPTT) as the SP-A isotherms of swollen DNA/Ca^2+^/mGel− overlap with Ca^2+^/mGel−. Certainly, spontaneous adsorption of mGel− evidences faster adsorption kinetics of Ca^2+^/Gel–, above and below the VPPT, which is maybe caused by conformational change inducing an increased hydrophobicity of the mGel− in the presence of Ca^2+^. Moreover, the dilatational elasticity also suggests an increased flattening and interpenetration of shells occurring in lower concentrations of mGel− in the presence of Ca^2+^. It is hypothesized that this increased hydrophobicity of swollen Ca^2+^/mGel− could affect the interaction with DNA, being less noticeable for shrunken Ca^2+^/mGel−, which is already made more hydrophobic by deswelling. Results presented here offer several open questions which deserve attention in order to understand the complexation of DNA with mGel−. Unravelling the role of electrostatics and specific interactions by bridging agents seem important aspects to consider in the future.

## 4. Materials and Methods

### 4.1. Materials

N-Isopropylacrylamide, NIPAM (2210-25-5) was purchased from Sigma-Aldrich (Madrid, Spain). Methoxypolyethylene glycol 750-methacrylatem 50% in water, MPEG750MA (26915-72-0), methacrylic acid, MAA (79-41-4), [2-(Methacryloyloxy)ethyl]trimethylammonium chloride, MATMAC (5039-78-1), and Ethylene glycol dimethacrylate, EGDMA (97-90-5) were all purchased from EVONIK (Darmstadt, Germany). Sodium persulfate, NaPS (7775-27-1) was purchased from BRENNTAG (Barcelona, Spain) and 2,2′-Azobis(2-methylpropionamidine)dihydrochloride, V-50 (2997-92-4) was provided by FUJIFILM (Barcelona, Spain). Double-stranded DNA, deoxyribonucleic acid sodium salt from calf thymus, type I (>98% purity, 89380), and Ca(NO_3_)_2_ (ACS reagent, 31218) were purchased from Sigma-Aldrich (Madrid, Spain). Deionized ultrapure water (0.054 μS) was from Milli-Q plus water purification system (Millipore, Madrid, Spain). All glassware was cleaned with 10% Micro-90^®^ cleaning solution, isopropanol (98% purity), and then repeatedly rinsed with distilled and ultrapure water.

### 4.2. Synthesis of Microgels

The microgel dispersions were prepared by a single-step surfactant-free batch precipitation polymerization reaction using the following recipe with reagents shown in Table 1 and the procedure described elsewhere [68]. The reaction mixture, consisting of water and monomers, including cross-linker (Table 1), was stirred for 20 min at 250 rpm with a nitrogen purge to remove dissolved oxygen. Once the reaction temperature had been reached, the initiator (Table 1) dissolved in 20 mL of water was added in one shot to the reactor and the polymerization was allowed to continue for 24 h at 70 °C. MPEG750MA is a methacylate monomer with 17 units of ethylene glycol. This methacrylate part is polymerized together with NIPAM and the rest of the comonomers forming a copolymer. The conversion of MATMAC and MAA is considered to be total in the reaction while the presence of crosslinker promotes interconnections between polymer chains and the formation of a gel inside the particles. The microgel was allowed to cool and then filtered through glass wool. The amounts of reagents used are detailed for cationic (mGel+) and anionic (mGel−) microgels as displayed in Table 1.

### 4.3. Hydrodynamic Diameter and Electrophoretic Mobility

A Zetasizer Nano ZS system from Malvern Instruments (Madrid, Spain), was used to measure both the hydrodynamic diameter (D_h_) and the electrophoretic mobility (µ_e_) of microgel particles in solution. D_h_ is obtained from the diffusion coefficient measured by the dynamic light scattering (DLS) technique from measurements of intensity correlation function and the so-called cumulant method at a scattering angle of 90°. According to this procedure, the logarithm of the intensity correlation function is fitted by a polynomial of third degree. The decay rate of this curve is proportional to the translational diffusion coefficient of the particles. Then, D_H_ is calculated by using the Stokes–Einstein equation and assuming that particles are spherical [69]. µ_e_ is defined as the rate of migration of charged particles induced by external electric field strength and related to the effective charge of a particle immersed in a solution. Samples were diluted 1:20 with ultrapure water and were dispersed by ultrasound around 30 s to avoid aggregation, assuring that no temperature change was recorded in this time scale of ultrasound. Measurements of D_h_ and µ_e_ of microgels as a function of the temperature were taken every 2 °C, ranging from 20 °C to 50 °C, in order to consider the whole variety of sizes of the microgels. µ_e_ of mGel+ was measured as a function of microgel concentration (0.025–1.5 g L^−1^) in the presence of [DNA] = 0.025 g L^−1^ and µ_e_ of mGel− was measured as a function of temperature in the presence of [Ca(NO_3_)_2_] = 1 mM. Mixtures of mGel and DNA were prepared at 20 °C and then taken to 50 °C. For each sample, six independent measurements were performed, and their corresponding standard deviations were calculated; plotted values are mean values with the standard deviations.

### 4.4. Langmuir Monolayers

Langmuir–Pockels film balance of a total area of 244.5 cm^2^ was used to form and measure the monolayers of microgel particles. This technique uses a paper Wilhelmy plate pressure measuring system (KSV) with a sensitivity of 0.1 mN m^−1^ to measure the surface pressure (SP) for variable surface area (A). The whole setup was located in a transparent Plexiglas case to avoid airstreams and dust deposition and was thermostatically controlled by an external thermostat. The microgel samples were incubated at the required temperature for 15 min before spreading on the surface. Then, the microgel suspension was carefully spread on the subphase (which was kept at the required temperature), employing a microsyringe (Hamilton). After 15 min for equilibration, the surface pressure area (SP-A) isotherm was recorded upon symmetric uniaxial compression at a constant rate of 10 mm min^−1^. Aqueous solutions with different solid masses were spread to cover the whole compression isotherm in each case. Solid masses ranging from 0.3 to 0.5 mg were spread from suspensions of mGel+ (9.95 g L^−1^) while solid masses ranging from 0.1 to 0.2 mg were spread from suspensions of mGel− (9.5 g L^−1^). SP-A isotherms were recorded separately at 20 °C and 45 °C to account for the different swelling states of microgels. For mGel+, the SP-A isotherms were recorded on ultrapure water subphase and on subphase containing [DNA] = 0.025 g L^−1^. For mGel−, the SP-A isotherms were recorded on ultrapure water subphase, and on subphases containing [Ca(NO_3_)_2_] = 1 mM, [DNA] = 0.025 g L^−1^, and both [Ca(NO_3_)_2_] + [DNA] together. The absence of surface-active contaminants was verified by compressing the pure water subphase, DNA, and Ca^2+^ solutions before every set of experiments, obtaining values of SP < 0.2 mN m^−1^ within the whole compression cycle. All isotherms were expressed in terms of SP-A/mass of mGel. Experiments were repeated at least 3 times for different samples and the standard deviation was less than 5%. Values plotted are mean values and replicates with standard deviations.

### 4.5. Adsorbed Monolayers

Adsorbed microgel films at the air-water interface were measured by a pendant drop surface film balance fully designed and assembled by the University of Granada (WO2012080536-A1) and described in detail elsewhere [59]. The whole of the equipment is computer-controlled by software DINATEN^®^ and the images are analyzed by software CONTACTO^®^. The detection and calculation of surface area (*A*) and surface tension (ST) are based on axisymmetric drop shape analysis (ADSA). A solution droplet is formed at the tip of the double capillary and the ST is recorded in real time at constant surface area *A* = 20 mm^2^ for 1 h or until stabilization of the surface film given by negligible variation of ST. Then, the surface dilatational viscoelastic modulus (*E*) is measured by oscillating of the drop volume and recording the response of the ST to the deformation of the adsorbed film:

(1) $$\left|E\right|=A\frac{d\gamma }{dA}$$

where *γ* is the surface tension.

*E* is therefore a complex quantity that contains a real (*E*′) and an imaginary part (*E*″):

(2) $$E^{\prime }=\left|E\right|\cos\delta ,E\prime \prime =\left|E\right|\sin\delta$$

where *δ* is the phase angle difference between the surface tension and surface area. *E*′ is the storage modulus that accounts for the surface elasticity (ε) of the adsorbed layer, *E*″ is the loss modulus that accounts for the surface viscosity (*η*) of the surface layer: $E=E^{\prime }+E^{\prime \prime }i=\varepsilon +i\eta \nu$, where ν is the angular frequency of the applied oscillation. The applied volume oscillations are maintained at amplitude values of less than 5%, to avoid excessive perturbation of the interfacial layer and the frequency can be varied between 0.01 and 1 Hz. Here, the frequency has been set to 0.1 Hz; at this relatively high frequency, the response of the adsorbed layer is mainly elastic so that the imaginary part has been neglected in the analysis and the complex dilatational modulus equals the storage modulus or surface dilatational elasticity [37].

The pendant drop is kept in a glass cuvette (Hellma^®^) located in a thermostatically controlled cell which is adjusted with an external temperature control to 20 °C and 45 °C to account for the swelling state of microgels. The temperature was measured inside the glass cuvette, close to the pendant drop and was checked during each experiment. The ST of the clean air-water surface is measured before every experiment to confirm the absence of surface-active contaminants, yielding values of (72.8 ± 0.2) mN m^−1^ at 20 °C and (68.8 ± 0.2) mN m^−1^ at 45 °C. Microgel solutions were prepared by dilution of mGel− in an aqueous subphase and incubation at the required temperature for 15 min before adsorption. Adsorption of mGel− was recorded for different concentrations of microgel ranging between 10^−5^ and 1 g L^−1^ in pure water subphase and in subphase containing [Ca(NO_3_)_2_] = 1 mM. All the measurements were carried out in triplicate, obtaining different standard deviations; plotted values are mean values with the standard deviations.

### 4.6. Statistical Analysis

Statgraphics 18 (Statistical Graphics Corp., Rockville, MD, USA) free version was used for statistical data analysis. Data are expressed as mean ± standard deviation. First, ANOVA was assayed with *p*-value < 0.001, followed by performance of Tukey´s multiple sample comparison analysis to identify significant differences between data. Differences between mean values were considered significant at a level of confidence of 95% (*p* < 0.05).

## Figures and Tables

**Table 1:** Reagents and amounts used in the recipe for cationic and anionic microgels.

	mGel+		mGel−	
Function	Reagent	Weight (g)	Reagent	Weight (g)
Solvent	Ultrapure water	1800	Ultrapure water	1800
Thermoresponsive monomer	NIPAM	14.4	NIPAM	14.4
PEGylated monomer	MPEG750MA	3.6	MPEG750MA	3.6
Ionic-comonomer	MATMAC *	3.6	MAA **	1.8
Crosslinker	EGDMA	0.9	EGDMA	0.9
Initiator	V-50 *	0.36	NaPS **	0.36

* Positive charge; ** negative charge.

## Data Availability

Not applicable.
